# Comparison of Swine and Human Computational Hemodynamics Models for the Study of Coronary Atherosclerosis

**DOI:** 10.3389/fbioe.2021.731924

**Published:** 2021-08-02

**Authors:** Giuseppe De Nisco, Claudio Chiastra, Eline M. J. Hartman, Ayla Hoogendoorn, Joost Daemen, Karol Calò, Diego Gallo, Umberto Morbiducci, Jolanda J. Wentzel

**Affiliations:** ^1^ PoliTo^BIO^Med Lab, Department of Mechanical and Aerospace Engineering, Politecnico di Torino, Turin, Italy; ^2^ Department of Cardiology, Biomedical Engineering, Erasmus MC, Rotterdam, Netherlands

**Keywords:** coronary artery disease, computational fluid dynamics, patient-specific modeling, wall shear stress, helical flow

## Abstract

Coronary atherosclerosis is a leading cause of illness and death in Western World and its mechanisms are still non completely understood. Several animal models have been used to 1) study coronary atherosclerosis natural history and 2) propose predictive tools for this disease, that is asymptomatic for a long time, aiming for a direct translation of their findings to human coronary arteries. Among them, swine models are largely used due to the observed anatomical and pathophysiological similarities to humans. However, a direct comparison between swine and human models in terms of coronary hemodynamics, known to influence atherosclerotic onset/development, is still lacking. In this context, we performed a detailed comparative analysis between swine- and human-specific computational hemodynamic models of coronary arteries. The analysis involved several near-wall and intravascular flow descriptors, previously emerged as markers of coronary atherosclerosis initiation/progression, as well as anatomical features. To do that, non-culprit coronary arteries (18 right–RCA, 18 left anterior descending–LAD, 13 left circumflex–LCX coronary artery) from patients presenting with acute coronary syndrome were imaged by intravascular ultrasound and coronary computed tomography angiography. Similarly, the three main coronary arteries of ten adult mini-pigs were also imaged (10 RCA, 10 LAD, 10 LCX). The geometries of the imaged coronary arteries were reconstructed (49 human, 30 swine), and computational fluid dynamic simulations were performed by imposing individualized boundary conditions. Overall, no relevant differences in 1) wall shear stress-based quantities, 2) intravascular hemodynamics (in terms of helical flow features), and 3) anatomical features emerged between human- and swine-specific models. The findings of this study strongly support the use of swine-specific computational models to study and characterize the hemodynamic features linked to coronary atherosclerosis, sustaining the reliability of their translation to human vascular disease.

## Introduction

Coronary atherosclerosis is a major cause of morbidity and mortality in Western World (Virani et al., 2021), consisting of the build-up of an atherosclerotic plaque in the wall of coronary arteries, possibly leading to severe stenosis and/or thrombus formation, with vascular lumen occlusion and death (Lusis, 2000).

The natural history of the coronary atherosclerotic disease is driven by a complex interplay of several local biological, systemic and biomechanical factors, with a still incomplete understanding of the underlying mechanisms (Chatzizisis et al., 2007; Wentzel et al., 2012; Kwak et al., 2014; Morbiducci et al., 2016; Zaromytidou et al., 2016). Although several large *in vivo* human studies have provided valuable insights into the initiation and progression of coronary atherosclerosis (Stone et al., 2012; Cheng et al., 2014), these studies remain often limited to short durations and limited number of imaging moments, mainly due to ethical reasons (Millon et al., 2014). Therefore, they lack the time scales necessary for the development of this pathology and might not capture all phenomena present during this complex, multifactorial disease (Daugherty et al., 2017). Such limitations may affect the *in vivo* investigation of innovative clinical strategies for coronary atherosclerotic treatment in humans (Xiangdong et al., 2011; Millon et al., 2014; Daugherty et al., 2017).

Animal models of coronary atherosclerosis have the potential to overcome many of these inherent restrictions of human studies (Xiangdong et al., 2011; Millon et al., 2014; Daugherty et al., 2017), facilitating the analysis of the coronary atherosclerotic disease at different stages (Daugherty et al., 2017). For this reason, several large animal models based on the use of rabbits, pigs, or non-human primates, have been adopted to e.g.: 1) study coronary atherosclerosis natural history (Getz and Reardon, 2012; Daugherty et al., 2017); 2) evaluate the efficacy of clinical treatment procedures (Shin et al., 2021); 3) identify predictive tools for the evolution of the disease, which most of the time is asymptomatic (De Nisco et al., 2020; Hoogendoorn et al., 2020; Mazzi et al., 2021). Most of the animal model-based studies on atherosclerosis onset and progression imply that their findings reliably inform human studies, sometimes suggesting a direct translation to humans. However, the value of animal models in identifying hemodynamic factors involved in the atherosclerotic disease or in predicting the effectiveness of treatment strategies in clinical trials has remained not fully clarified.

In relation to coronary atherosclerotic disease, the capability of an animal model to mimic the complexity of the human coronary pathophysiology plays a critical role (Daugherty et al., 2017; Shin et al., 2021). In this regard, due to their similarities to humans in terms of anatomy (i.e., size and distribution) (Weaver et al., 1986; Lelovas et al., 2014), pathophysiology (Lelovas et al., 2014; Shim et al., 2016), lipoprotein profile (Mahley et al., 1975), and site of lesion formation (Shim et al., 2016), swine models are massively applied to study coronary atherosclerosis (Xiangdong et al., 2011; Daugherty et al., 2017). In particular, swine models of familial hypercholesterolemia with a mutation in genes coding for apolipoproteins and low-density lipoproteins receptor allows studying sustained atherosclerotic plaques onset/progression because of their capability to develop advanced atherosclerotic lesions within 12–18 months when fed a high fat diet (Thim et al., 2010; Daugherty et al., 2017).

Besides the already investigated differences/similarities between human and swine models, a direct comparison in terms of coronary hemodynamics, a well-established biomechanical factor influencing atherosclerotic onset/development (Wentzel et al., 2012; Morbiducci et al., 2016), is still lacking. This may limit the translation to human models of previous findings suggesting the role of near-wall and intravascular hemodynamic descriptors as markers of coronary atherosclerosis initiation/progression in swine-specific models (Chatzizisis et al., 2008; De Nisco et al., 2019, 2020; Hoogendoorn et al., 2020).

Aiming to bridge this gap of knowledge, in this study we perform, for the first time, a detailed comparative analysis between swine- and human-specific computational hemodynamic models of the three main coronary arteries, in terms of several descriptors of 1) near-wall and 2) intravascular flow quantities, and 3) vessel morphology, that have been already identified as biomechanical risk factors in the initiation/progression of coronary atherosclerotic plaques (Stone et al., 2012; Wentzel et al., 2012).

## Materials and Methods

### Human Population

Forty-eight hemodynamically stable patients from the IMPACT study data set (Hartman et al., 2020) were involved in the analysis. Clinical characteristics are listed in Table 1. The IMPACT study enrolled patients with acute coronary syndrome and with at least one non-stented non-culprit coronary segment accessible for intracoronary imaging study. The presence of previous coronary artery bypass graft surgery, 3-vessel disease, renal insufficiency (creatinine clearing <50 ml/min), left ventricular ejection fraction <30%, and atrial fibrillation, were considered as exclusion criteria. All patients underwent percutaneous coronary intervention of the culprit coronary vessel. After successful treatment, a non-culprit coronary segment (right–RCA, left anterior descending–LAD, or left circumflex–LCX coronary artery) was imaged and used for the study.

**TABLE 1:** **-** Human dataset clinical characteristics.

Clinical characteristics	
N = 48 subjects	
Age (years)	61.3 ± 9
Men (%)	43 (90%)
Body mass index	27.0 ± 4.5
Diabetes mellitus, n (%)	8 (17%)
Hypertension, n (%)	14 (29%)
Dyslipidemia, n (%)	23 (48%)
Smokers, n (%)	36 (75%)
Positive family history, n (%)	19 (40%)
Previous MI, n (%)	9 (19%)
Previous PCI, n (%)	11 (23%)
LDL (mmol/L)	2.84 ± 1.02

MI: myocardial infarction; PCI: percutaneous coronary intervention; LDL: low-density lipoproteins.

All patients gave their informed consent. The study was approved by the local medical ethical committee of the Erasmus MC (MEC 2015-535, NL54519.078.15), was registered (ISCRTN:43,170,100) and conducted in accordance with the World Medical Association Declaration of Helsinki (64th WMA General Assembly, Fortaleza, Brazil, October 2013) and Medical Research Involving Human Subject Act (WMO).

### Animal Model

Ten adult familial hypercholesterolemia Bretoncelles Meishan mini-pigs with a low-density lipoprotein receptor mutation were enrolled in the analysis. The study involved the three main coronary arteries (i.e., RCA, LAD, and LCX) of each animal at 3 months after the start of a high fat diet. At this stage, the animals were considered ostensibly healthy.

The study was approved by the local animal ethics committee of the Erasmus MC (EMC nr. 109-14-10) and performed according to the National Institute of Health guide for the Care and Use of Laboratory animals (Council, 2011).

### Medical Imaging and Geometry Reconstruction

**FIGURE 1:**
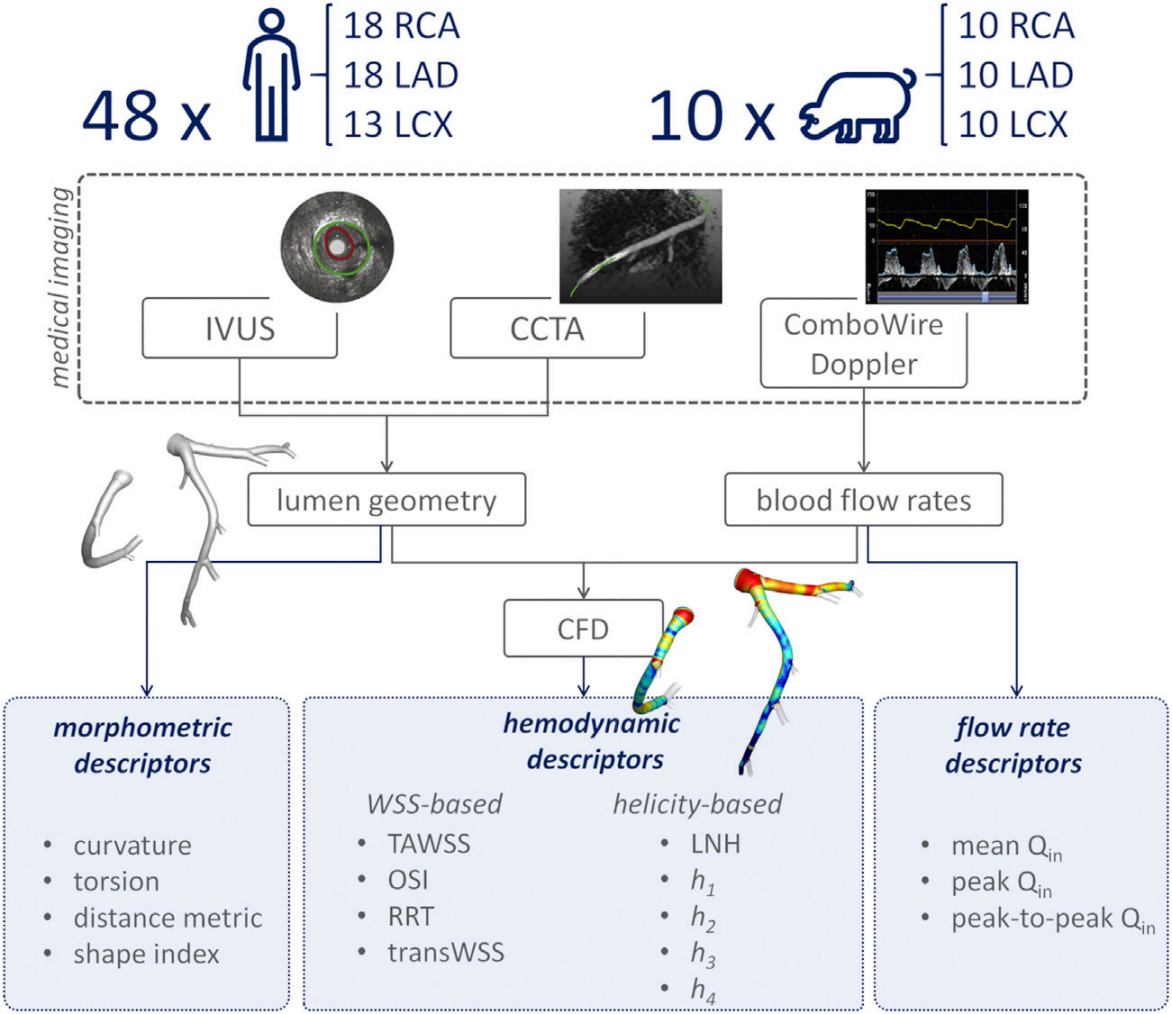
Schematic diagram of the study design, showing how imaging data contribute to define vessel geometry and hemodynamic, morphometric, and flow variables. RCA, LAD, and LCX denote right, left anterior descending, and left circumflex coronary artery, respectively. IVUS: intravascular ultrasound; CCTA: coronary computed tomography angiography; CFD: computational fluid dynamics; TAWSS: time-averaged wall shear stress; OSI: oscillatory shear index; RRT: relative residence time; transWSS: transverse wall shear stress; LNH: local normalized helicity; *h*_*1*_: average helicity; *h*_*2*_: average helicity intensity; *h*_*3*_: signed balance of counter rotating helical flow structures; *h*_*4*_: unsigned balance of counter rotating helical flow structures; Q_in_: inflow rate.

**FIGURE 2:**
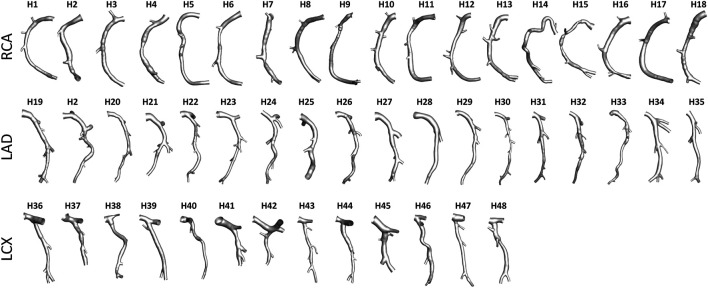
Geometry of the 49 human coronary artery models. Labels from H1 to H48 identify the single patient. Right (RCA), left anterior descending (LAD), and left circumflex (LCX) coronary artery geometries are grouped.

**FIGURE 3:**
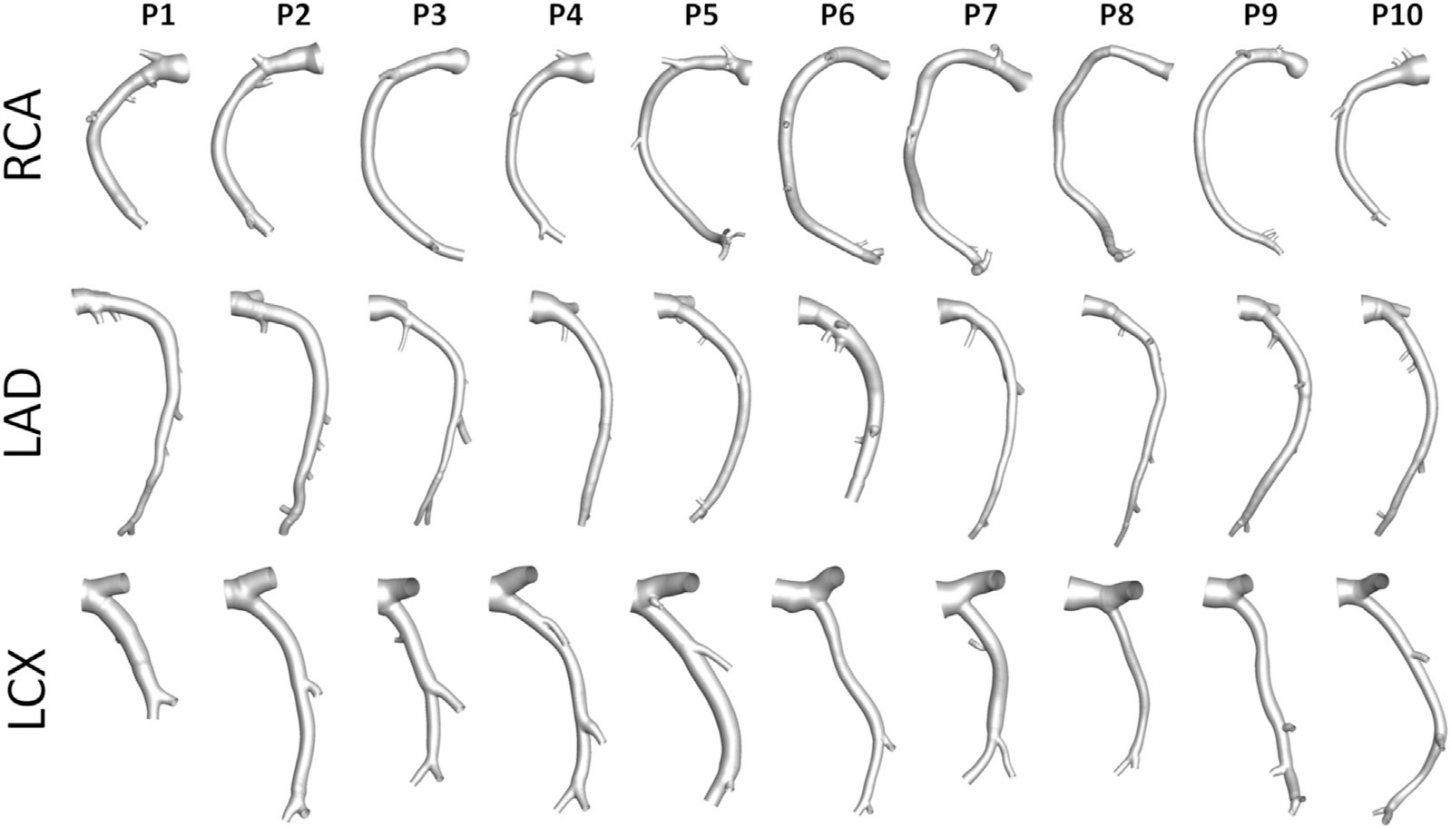
Geometry of the 30 swine coronary artery models. Labels from P1 to P10 identify the single pig. Right (RCA), left anterior descending (LAD), and left circumflex (LCX) coronary artery geometries are grouped.

An overview of the methods is provided in Figure 1. The same imaging protocol was applied to human- and swine-specific coronary segments. Each coronary artery was imaged by computed coronary tomography angiography (CCTA) (SOMATOM Force, Siemens Healthineers, Germany) and intravascular ultrasound (IVUS) (InfraRedX, Burlington, MA, United States), as detailed elsewhere (De Nisco et al., 2019, 2020; Hartman et al., 2020; Hoogendoorn et al., 2020). Coronary lumen contours were segmented on IVUS images (QCU-CMS, Medis Medical Imaging, Leiden) and aligned along the 3D CCTA centerline in order to reconstruct the 3D vessel geometry. Additional luminal regions proximally (up to the aorta) and at least two diameters distally to the IVUS-imaged segment were reconstructed using CCTA images (De Nisco et al., 2019, 2020; Hartman et al., 2020; Hoogendoorn et al., 2020). The 79 reconstructed luminal surfaces of the coronary arteries (49 human-specific models: 18 RCA, 18 LAD, and 13 LCX; 30 swine-specific models: 10 RCA, 10 LAD, and 10 LCX) are presented in Figure 2 and Figure 3 for humans and animal models, respectively. The 3D geometries were reconstructed including the side branches.

Combowire Doppler (Phillips Volcano, Zaventem, Belgium) flow velocity measurements were acquired in each coronary artery at the inflow section and immediately upstream and downstream of each side branch, as extensively detailed elsewhere (De Nisco et al., 2019, 2020; Hartman et al., 2020; Hoogendoorn et al., 2020).

### Computational Hemodynamics

The reconstructed vessel geometries were discretized, and unsteady-state computational fluid dynamics (CFD) simulations were performed to characterize coronary hemodynamics (Figure 1). The governing equations of fluid motion were numerically solved in Fluent environment (ANSYS Inc. Canonsburg, PA, United States of America), by using the finite volume method. All the CFD settings are extensively detailed elsewhere (De Nisco et al., 2019, 2020; Hartman et al., 2020; Hoogendoorn et al., 2020). Briefly, blood was assumed as an incompressible, homogeneous, non-Newtonian fluid (Chiastra et al., 2017). No-slip condition was assumed at the arterial wall. *In vivo* ComboWire Doppler velocity measurements were used to derive individualized (specific for each human and swine model, as reported elsewhere (De Nisco et al., 2019, 2020; Hartman et al., 2020; Hoogendoorn et al., 2020)) boundary conditions (BCs) as follows: 1) the inlet flow rate was estimated from the most proximal Doppler velocity measurement, and prescribed as inlet boundary condition in terms of time-dependent flat velocity profile; 2) side branches perfusion was quantified as the difference between upstream and downstream Doppler velocity-based flow rate measurements and applied as outflow condition in terms of measured flow ratio. If velocity-based flow measurements were inaccurate or not available, a generalized flow rate (van der Giessen et al., 2011) was prescribed as inflow BC, while a proper diameter-based scaling law for human- (van der Giessen et al., 2011) and swine-specific (Huo and Kassab, 2012) models was applied to estimate the flow ratio at the outflow section (De Nisco et al., 2019, 2020; Hartman et al., 2020; Hoogendoorn et al., 2020).

### Hemodynamic Descriptors

Near-wall and intravascular hemodynamics were analyzed by computing the hemodynamic quantities listed in Table 2.

**TABLE 2:** **-** Definition of hemodynamic descriptors involved in the analysis.

WSS-based descriptors	
Time-Averaged WSS (TAWSS)	$\text{TAWSS}=\frac{1}{T}\overset{T}{\underset{0}{\int }}\left|\mathrm{WSS}\right|dt$
Oscillatory Shear Index (OSI)	$\text{OSI}=0.5\;\left[1-\left(\frac{\left|\int_{0}^{T}\mathrm{WSS}dt\right|}{\int_{0}^{T}\left|\mathrm{WSS}\right|dt}\right)\right]$
Relative Residence Time (RRT)	$\text{RRT}=\frac{1}{\text{TAWSS}\cdot \left(1-2\cdot \text{OSI}\right)}=\frac{1}{\frac{1}{T}\left|\int_{0}^{T}\mathrm{WSS}dt\right|}$
Transverse WSS (transWSS)	$\text{transWSS}=\frac{1}{T}\overset{T}{\underset{0}{\int }}\left|\mathrm{WSS}\cdot \left(n\times \frac{\int_{0}^{T}\mathrm{WSS}dt}{\left|\int_{0}^{T}\mathrm{WSS}dt\right|}\right)\right|dt$
**Helicity-based descriptors**
Local Normalized Helicity (LNH)	$\text{LNH}=\frac{v\cdot \omega }{\left|v\right|\cdot \left|\omega \right|}=\cos\gamma$
Average helicity ($h_{1}$)	$h_{1}=\frac{1}{TV}\iint_{T\;V}v\cdot \omega \;dV\;dt$
Average helicity intensity ($h_{2}$)	$h_{2}=\frac{1}{TV}\iint_{T\;V}\left|v\cdot \omega \right|\;dV\;dt$
Signed balance of counter-rotating helical flow structures ($h_{3}$)	$h_{3}=\frac{h_{1}}{h_{2}}\;\;\;\;\;\;\;\;\;\;\;\;-1\;\leq h_{3}\leq 1$
Unsigned balance of counter-rotating helical flow structures ($h_{4}$)	$h_{4}=\frac{\left|h_{1}\right|}{h_{2}}\;\;\;\;\;\;\;\;\;\;\;\;\;\;0\;\leq h_{4}\leq 1$

***WSS*** is the WSS vector; T is the period of the cardiac cycle; ***n*** is the unit vector normal to the arterial surface at each element; ***v*** is the velocity vector; ***ω*** is the vorticity vector; *γ* is the angle between velocity and vorticity vectors; V is the arterial volume.

Near-wall hemodynamics was characterized in terms of the three canonical WSS-based descriptors, namely time-averaged wall shear stress (TAWSS), oscillatory shear index (OSI) (Ku et al., 1985), and relative residence time (RRT) (Himburg et al., 2004). Additionally, the transversal WSS (transWSS) (Peiffer et al., 2013), a descriptor of WSS multidirectionality, was also considered. The transWSS represents the average WSS component acting orthogonal to the time-average WSS vector direction (Table 2).

Based on the recently-emerged atheroprotective role of physiological helical-shaped blood flow structures in coronary arteries (De Nisco et al., 2019, 2020), intravascular hemodynamics was investigated in terms of helical flow, quantified through the quantities summarized in Table 2. In detail, the local normalized helicity (LNH) (Morbiducci et al., 2007), representing the cosine of the angle between local velocity **(v)** and vorticity (**ω**) vectors (Table 2), was used to visualize right- and left-handed helical blood flow patterns (positive and negative LNH values, respectively) (Gallo et al., 2012; Morbiducci et al., 2013) inside the coronary artery models. Furthermore, four additional helicity-based descriptors (Gallo et al., 2012; Morbiducci et al., 2013) were applied to characterize the strength, size and relative rotational direction of helical flow in the 79 coronary artery models (Table 2): cycle-average helicity (*h*_*1*_) and helicity intensity (*h*_*2*_), indicating the net amount and the intensity of helical flow, respectively; signed (*h*_*3*_) and unsigned helical rotation balance (*h*_*4*_), measuring the prevalence (identified by the sign of descriptor *h*_*3*_) or only the strength of relative rotations of helical flow patterns, respectively.

The hemodynamics of each coronary vessel was characterized also in terms of inflow rate (Q_in_), as given by its mean, peak, and peak-to-peak values (Figure 1). Peak-to-peak Q_in_ was defined as the difference between the maximum and the minimum values of the inflow rate.

### Morphometry

**FIGURE 4:**
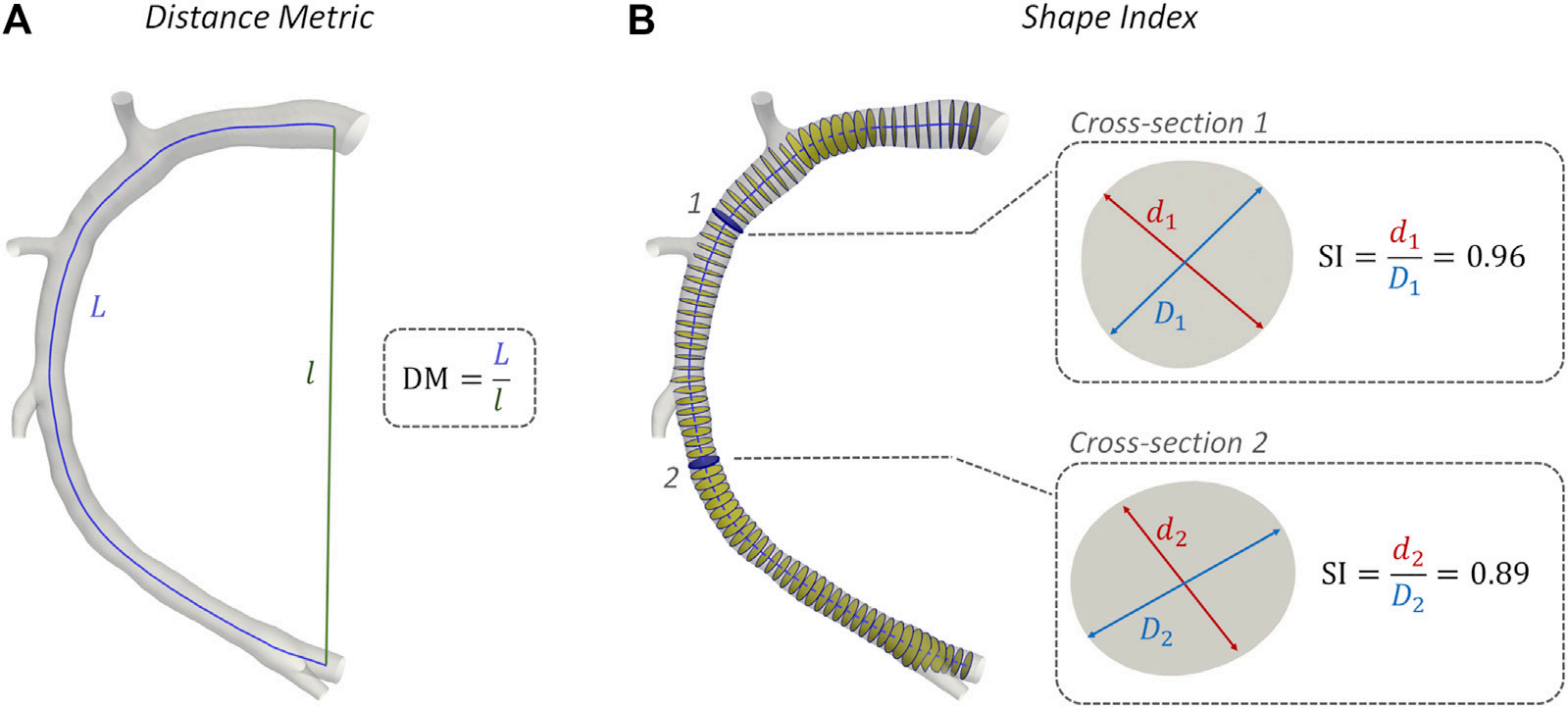
Methodology of Distance Metric (DM) and Shape Index (SI) assessment. Model H1 was taken as explanatory example. **(A)** DM computing: *L* and *l* are the curvilinear (blue) and Euclidean (green) distance between the centerline curve endpoints; **(B)** SI computation at two explanatory coronary cross-sections along the vessel centerline: *d* and *D* are the minimum and maximum diameter of arterial cross-section, respectively.

The geometric quantities summarized in Table 3 were adopted for characterizing coronary vessel morphometry. In detail, a robust centerline-based analysis was performed, where vessel curvature (κ) and torsion (τ) were assessed according to an approach proposed elsewhere (Gallo et al., 2015). Briefly, after extracting the main vessel centerline (defined as the geometrical locus of the centers of maximum inscribed spheres) in the Vascular Modeling Toolkit (VMTK, Orobix, Bergamo, Italy, http://www.vmtk.org/) environment, its continuous, noise-free analytical formulation (**C**) was obtained by adopting free-knots regression splines (Sangalli et al., 2009). Coronary curvature and torsion were then calculated by differentiation of the free-knots regression, spline centerline representation (Table 3). Here the average values of curvature ($\bar{\text{κ}}$) and torsion ($\bar{\text{τ}}$) along the main vessel were considered, which are known to have an influence on arterial hemodynamics (Gallo et al., 2015). Additionally, the degree of tortuosity of coronary vessels was assessed by computing the standard Distance Metric index (DM, Table 3) (Vorobtsova et al., 2016; Ciurică et al., 2019). DM, computed as the ratio between the curvilinear (*L*) and Euclidean (*l*) distance between the centerline curve endpoints (Figure 4A), quantifies the “lengthening effect” of coronary tortuosity. Finally, coronary cross-section eccentricity along the main vessel centerline was measured by computing the Shape Index (SI), as the ratio between the local cross-section minimum (*d*) and the maximum (*D*) diameter (Finotello et al., 2020). To calculate the SI, the opensource Vascular Modelling Toolkit software (VMTK, Orobix, Bergamo, Italy, http://www.vmtk.org/) was used. SI ranges between 0 and 1, where one indicates a perfectly circular cross-sectional shape (Figure 4B). Like for κ and τ, the average value of Shape Index ($\bar{\text{SI}}$) along the main vessel was considered for the analysis.

**TABLE 3:** **-** Definition of the geometric quantities adopted for characterizing coronary vessels morphometry.

Morphometric descriptors	
Curvature (κ)	$\text{κ}\left(s\right)=\frac{\left|C'\left(s\right)\times C''\left(s\right)\right|}{{\left|C'\left(s\right)\right|}^{3}}$
Torsion (τ)	$\text{τ}\left(s\right)=\frac{\left[C'\left(s\right)\times C''\left(s\right)\right]\cdot C'''\left(s\right)}{{\left|C'\left(s\right)\times C'\left(s\right)\right|}^{2}}$
Distance Metric (DM)	$\text{DM}=\frac{L}{l}\;\;\;\;\;\;\;\;\;\;\;\;\;\;\;\;\;\;\;\;\;\;\;\;\;\;\;\;\text{DM}\geq 1$
Shape Index (SI)	$\text{SI}\left(s\right)=\frac{d\left(s\right)}{D\left(s\right)}\;\;\;\;\;\;\;\;\;\;\;\;\;\;\;\;\;0\leq \text{SI}\leq 1$

**C′**, **C′′**, and **C‴** are the first, second, and third derivative of the centerline curve **C**, respectively; s is the curvilinear abscissa; L and I are the curvilinear and Euclidean distance between the centerline curve endpoints, respectively; d and D are the minimum and maximum diameter of arterial cross-section, respectively.

### Statistical Analysis

Data analysis was performed in the main branch of the RCA, LAD and LCX segments only, by removing coronary side branches in the post-processing step using VMTK. Hemodynamic and morphometric data were grouped according to the population (humans *vs* animals) and the coronary vessel type (i.e., RCA, LAD, or LCX). Differences between the two populations were investigated in Matlab environment (The MathWorks Inc. United States of America) by the Mann-Whitney *U*-test. Statistical significance was assumed for *p* < 0.05.

## Results

### Near-Wall and Intravascular Flow Features Visualization

**FIGURE 5:**
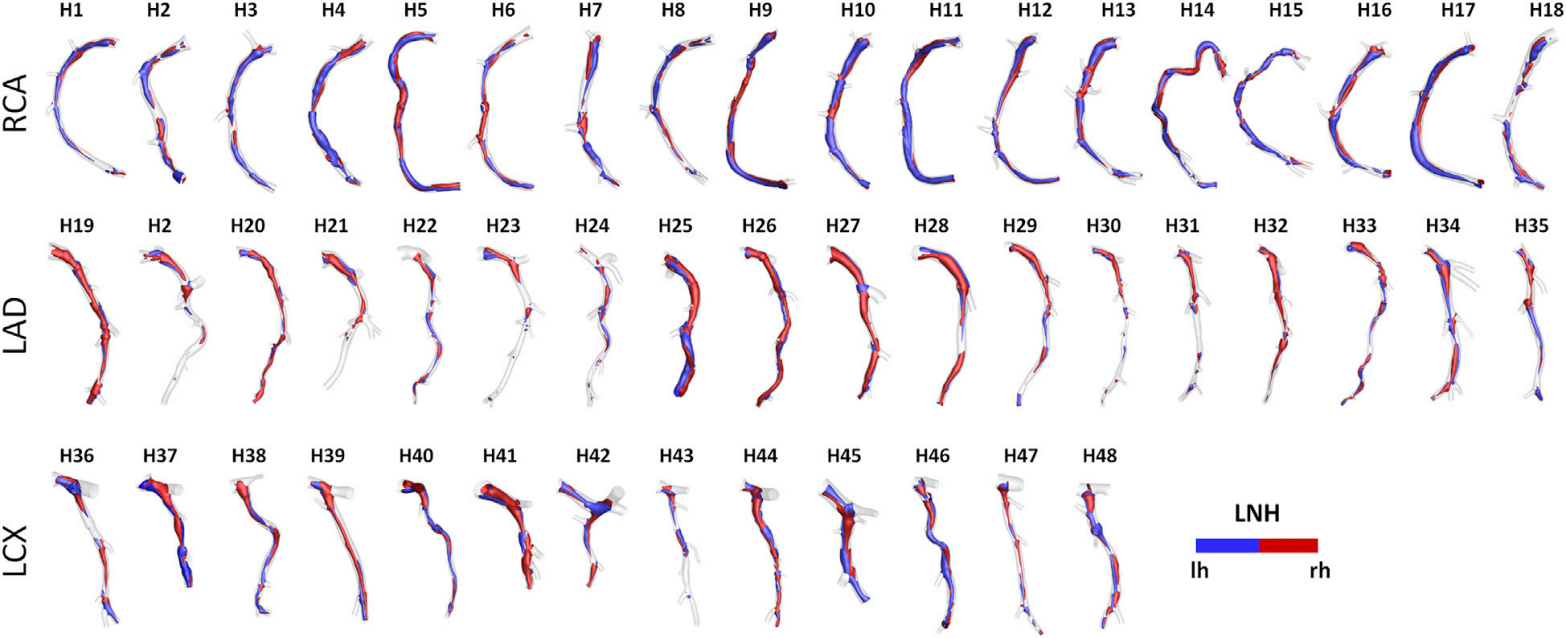
Intravascular fluid structures in the 49 human coronary arteries. For each case, isosurfaces of cycle-average LNH are presented. Distinguishable left-handed (lh - LNH < 0) and right-handed (rh - LNH > 0) helical flow structures can be observed in all coronary arteries. Labels from H1 to H48 identify the single patient. Right (RCA), left anterior descending (LAD), and left circumflex (LCX) coronary artery geometries are grouped.

**FIGURE 6:**
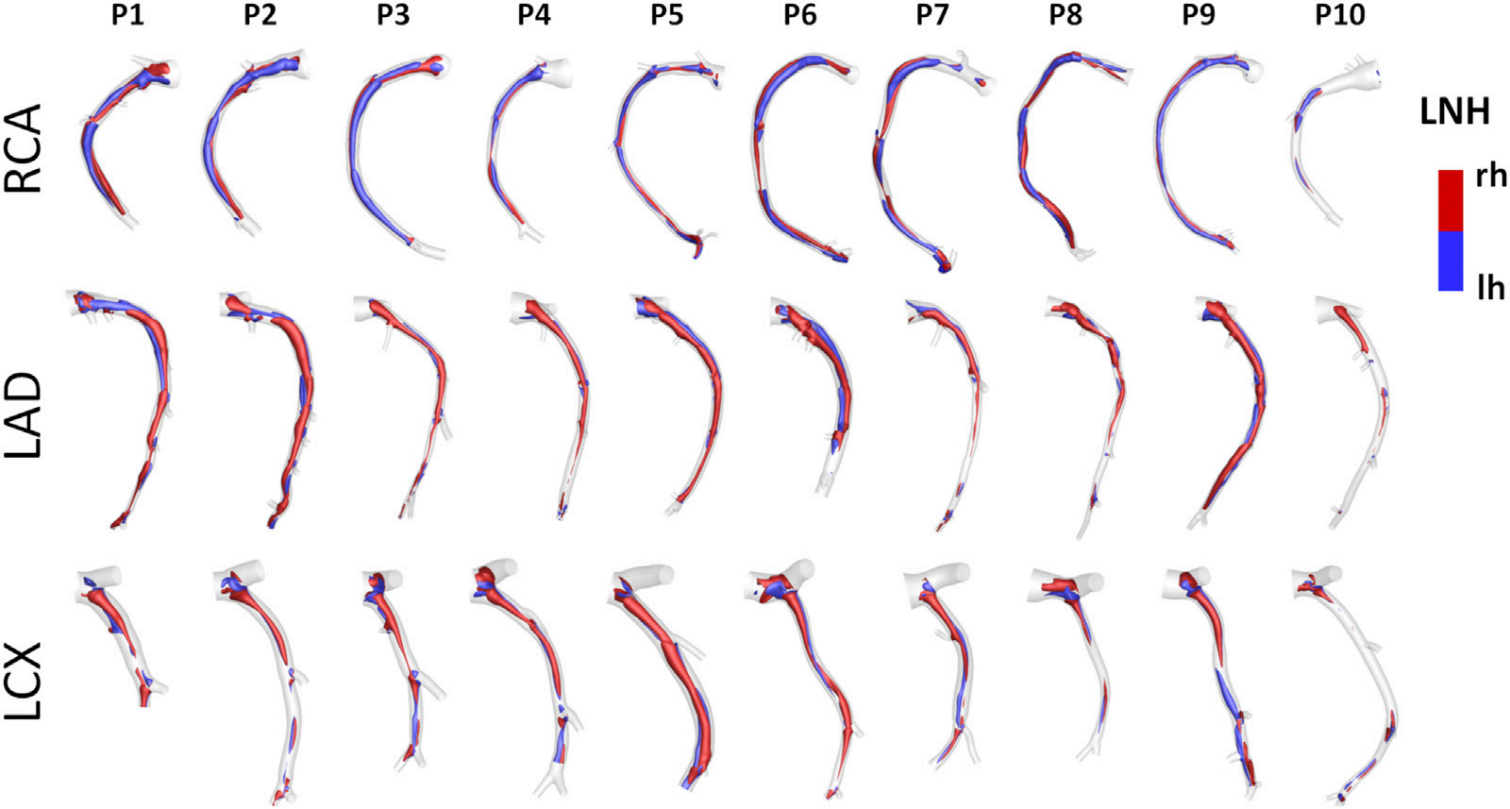
Intravascular fluid structures in the 30 swine coronary arteries. For each case, isosurfaces of cycle-average LNH are presented. Distinguishable left-handed (lh - LNH < 0) and right-handed (rh - LNH > 0) helical flow structures can be observed in all coronary arteries. Labels from P1 to P10 identify the single pig. Right (RCA), left anterior descending (LAD), and left circumflex (LCX) coronary artery geometries are grouped.

Helical blood flow patterns developing in human and swine coronary models were visualized in Figure 5 and Figure 6, respectively, using the cycle-average LNH isosurface values (blue and red colors indicate left-handed and right-handed helical flow patterns, respectively). Despite intra- and inter-species variations, the intravascular hemodynamics of both human and swine coronary arteries were markedly characterized by the presence of two distinguishable counter-rotating helical flow patterns.

**FIGURE 7:**
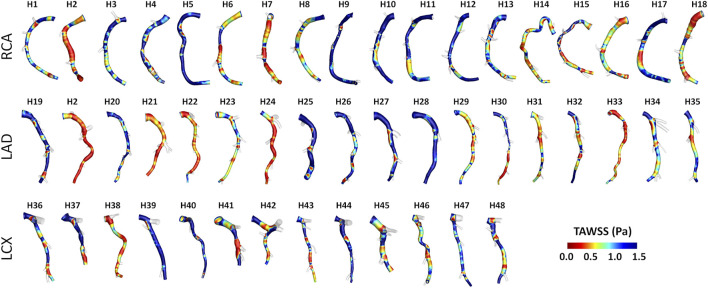
TAWSS distribution at the luminal surface of the 49 human coronary arteries. Red color highlights luminal regions exposed to low TAWSS values. Labels from H1 to H48 identify the single patient. Right (RCA), left anterior descending (LAD), and left circumflex (LCX) coronary artery geometries are grouped.

**FIGURE 8:**
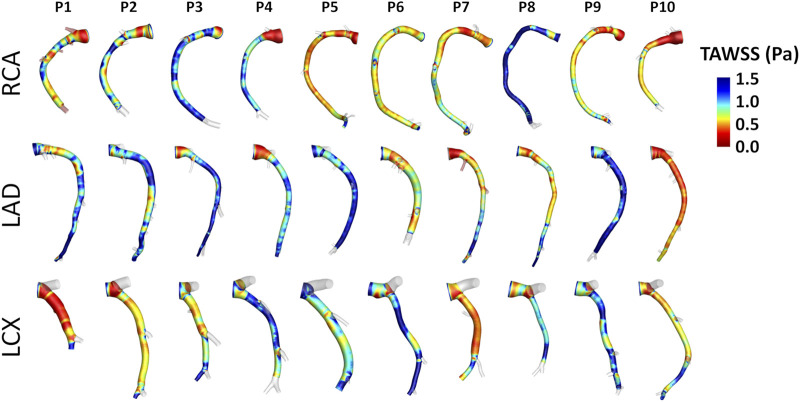
TAWSS distribution at the luminal surface of the 30 swine coronary arteries. Red color highlights luminal regions exposed to low TAWSS values. Labels from P1 to P10 identify the single pig. Right (RCA), left anterior descending (LAD), and left circumflex (LCX) coronary artery geometries are grouped.

Intra- and inter-species differences were analyzed by visual inspection also in terms of TAWSS luminal distribution, presented in Figure 7 and Figure 8 for human and swine models, respectively. In detail, the luminal surface of some of the human coronary arteries were largely exposed to low TAWSS values (red color in figure, e.g., cases H2-RCA, H21-LAD, and H38-LCX), whereas other human arteries were not (e.g., cases H5-RCA, H28-LAD, and H39-LCX). The same observations on intra-species variability can also be extended to swine models (Figure 8), with some individual cases exposed to low TAWSS values over most of the luminal surface (e.g., cases P5-RCA, P10-LAD, and P7-LCX) and other ones presenting with low TAWSS luminal regions of moderate extension (e.g., cases P8-RCA, P9-LAD, and P4-LCX).

### Hemodynamics: Quantitative Analysis

**FIGURE 9:**
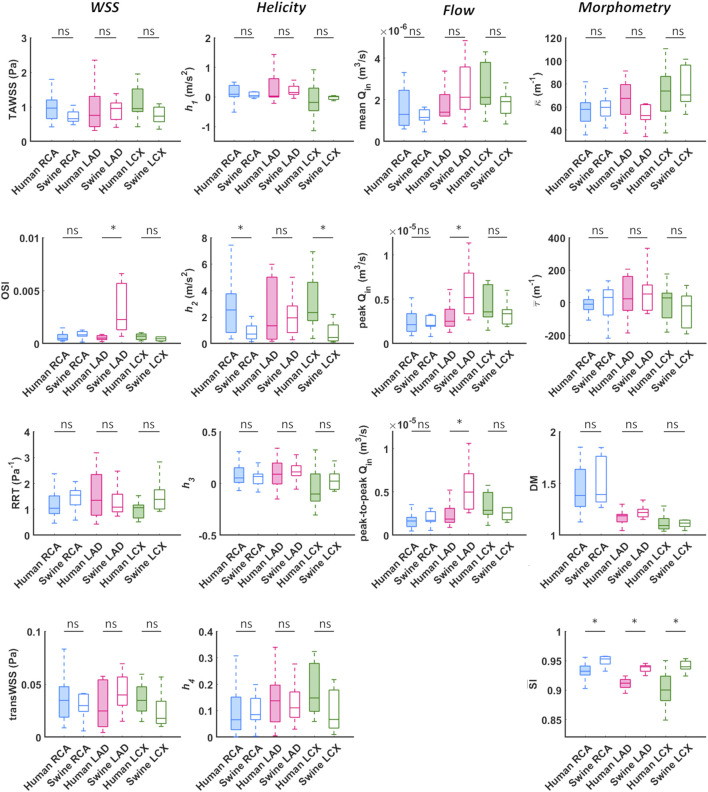
Box plots comparing WSS, helicity, flow rate, and morphometry in human and swine coronary arteries. For each population, right (RCA), left anterior descending (LAD), and left circumflex (LCX) coronary artery geometries are grouped and distinguished by box color (blue, magenta, and green, respectively). *WSS* column: TAWSS - time-average wall shear stress, OSI - oscillatory shear index, RRT - relative residence time, transWSS - transverse wall shear stress; *Helicity* column: *h*_*1*_ - average helicity, *h*_*2*_ - average helicity intensity, *h*_*3*_ - signed balance of counter rotating helical flow structures, *h*_*4*_ - unsigned balance of counter rotating helical flow structures; *Flow* column: Q_in_ - inflow rate; *Morphometry* column: κ - curvature, τ - torsion, DM - distance metric, SI - similarity index. Median and interquartile range are displayed for each descriptor. *p < 0.05.

A detailed quantitative comparison between human and swine models is presented in Figure 9 in terms of hemodynamics and morphometry. Human and swine models were grouped per species and per coronary vessel. Among the WSS-based descriptors, no significant difference emerged between the two species (*WSS* column in Figure 9) in terms of TAWSS (confirming the qualitative results presented in Figures 7, 8), RRT, and transWSS. Contrarily, a significant inter-species difference emerged for OSI but only for the LADs (*p* < 0.001), with swine LADs characterized by higher median value and larger interquartile range than human LADs (0.0005 [0.0004, 0.0007] and 0.0023 [0.0013, 0.0057] for human and swine models, respectively). Interestingly, an overall poor WSS multidirectionality emerged in both species, with OSI values lower than 0.08 (0.0005 [0.0003, 0.0009] and 0.0009 [0.0006, 0.0022] for human and swine models, respectively), and transWSS values lower than 0.10 Pa (0.03 [0.02, 0.05] and 0.03 [0.02, 0.04] Pa for human and swine models, respectively).

The quantitative analysis of the intravascular flow patterns, based on helicity-based descriptors (*Helicity* column in Figure 9), highlighted a significant inter-species difference for helical flow intensity in RCA (*p* < 0.05) and LCX (*p* < 0.01). In detail, human RCA and LCX vessels (2.53 [0.83, 3.76] and 2.33 [1.73, 4.63] m/s^2^ for human RCA and LCX, respectively) exhibited higher *h*_*2*_ values compared to the respective swine vessels (0.71 [0.35, 1.33] and 0.46 [0.20, 1.40] m/s^2^ for swine RCA and LCX, respectively), and larger intra-species variability. Of note, no significant inter-species difference emerged for topological quantities *h*_*3*_ and *h*_*4*_, suggesting an overall similar configuration of the counter-rotating helical flow patterns developing in human and swine coronary arteries, presented in Figures 5, 6.

The analysis of the hemodynamics is completed by the *Flow* column in Figure 9. Overall, human and swine coronary vessels were characterized by similar mean, peak, and peak-to-peak values of measured inflow rate, except for the LAD, where swine models presented significantly higher Q_in_ maximum values (2.52.10^−5^ [1.95.10^−5^, 3.87.10^−5^] and 5.20.10^−5^ [3.36.10^−5^, 7.95.10^−5^] m^3^/s for human and swine models, respectively; *p* < 0.05) and larger dynamics (1.86.10^−6^ [1.48.10^−6^, 3.09.10^−6^] and 4.97.10^−6^ [3.01.10^−6^, 7.09.10^−6^] m^3^/s for human and swine models, respectively; *p* < 0.01) than human models.

### Morphometry: Quantitative Analysis

No significant inter-species difference emerged in vessel curvature, torsion, and tortuosity (*Morphometry* column in Figure 9). In both populations, RCA models were characterized by higher DM values and larger intra-species variability, compared to LAD and LCX vessels. Conversely, a significant inter-species difference emerged in coronary vessels eccentricity, as measured by the shape index (*p* < 0.01). In detail, the cross-section of human coronary arteries presented with a more elliptical shape than swine arteries (Figure 4B and Figure 9), as highlighted by the lower $\bar{\text{SI}}$ values.

## Discussion

Swine models have contributed to a deeper understanding of the natural history of coronary atherosclerosis, with a valuable application to multiple research fields, such as the study of novel clinical treatment procedures efficacy (Getz and Reardon, 2012; Daugherty et al., 2017; Shin et al., 2021). The translation of results obtained in swine models to the patient situation is mainly based on intra-species comparisons of the coronary anatomy (Weaver et al., 1986; Lelovas et al., 2014), pathophysiology (Lelovas et al., 2014; Shim et al., 2016), cholesterol levels (Mahley et al., 1975) and sites of lesion formation (Shim et al., 2016). However, a direct comparison in terms of coronary local hemodynamics, a recognized risk factor of atherosclerosis (Chatzizisis et al., 2007; Wentzel et al., 2012; Kwak et al., 2014; Morbiducci et al., 2016; Zaromytidou et al., 2016), is still lacking.

Here a detailed comparative analysis between 49 human- and 30 swine-specific computational hemodynamic models of coronary arteries is presented in terms of coronary near-wall and intravascular hemodynamics, and morphometry. The study highlighted that overall human and swine coronary models present equivalent near-wall and intravascular hemodynamics, as well as equivalent geometrical features, with some minor exceptions, as discussed below.

### Hemodynamics

No significant inter-species difference emerged in terms of TAWSS, RRT, and transWSS in the three main coronary arteries. The two populations differed only in terms of OSI of the LAD coronary segment, with swine vessels presenting with significantly higher OSI median values than the human ones (0.0023 [0.0013, 0.0057] and 0.0005 [0.0004, 0.0007] for swine and human models, respectively, *p* < 0.001). Such inter-species difference can be interpreted as a consequence of the observed inter-species difference in the measured inflow rates at LAD coronary arteries, presenting with significantly larger peak and peak-to-peak values in swine than human models (*p* < 0.05 and *p* < 0.01, respectively), thus physically precipitating oscillations of the WSS vector along the cardiac cycle (Ku et al., 1985; Soulis et al., 2006; Gallo et al., 2018).

As previous research has shown (De Nisco et al., 2019, 2020), relatively low WSS multidirectionality is present in coronary arteries of both ostensibly healthy swine and human coronary arteries, as reflected by the observed very low OSI and transWSS values (*WSS* column in Figure 9). Even though low multidirectionality was observed, plaque progression was proven to be significantly related to multidirectional WSS parameters (Hoogendoorn et al., 2020). However, multidirectional WSS seems to be more relevant for atherosclerotic plaque development in later stages of the disease and therefore this inter-species observed difference in OSI in the LAD is of less relevance for studies on atherosclerotic plaque initiation (Gallo et al., 2018; Kok et al., 2019; Hoogendoorn et al., 2020).

**FIGURE 10:**
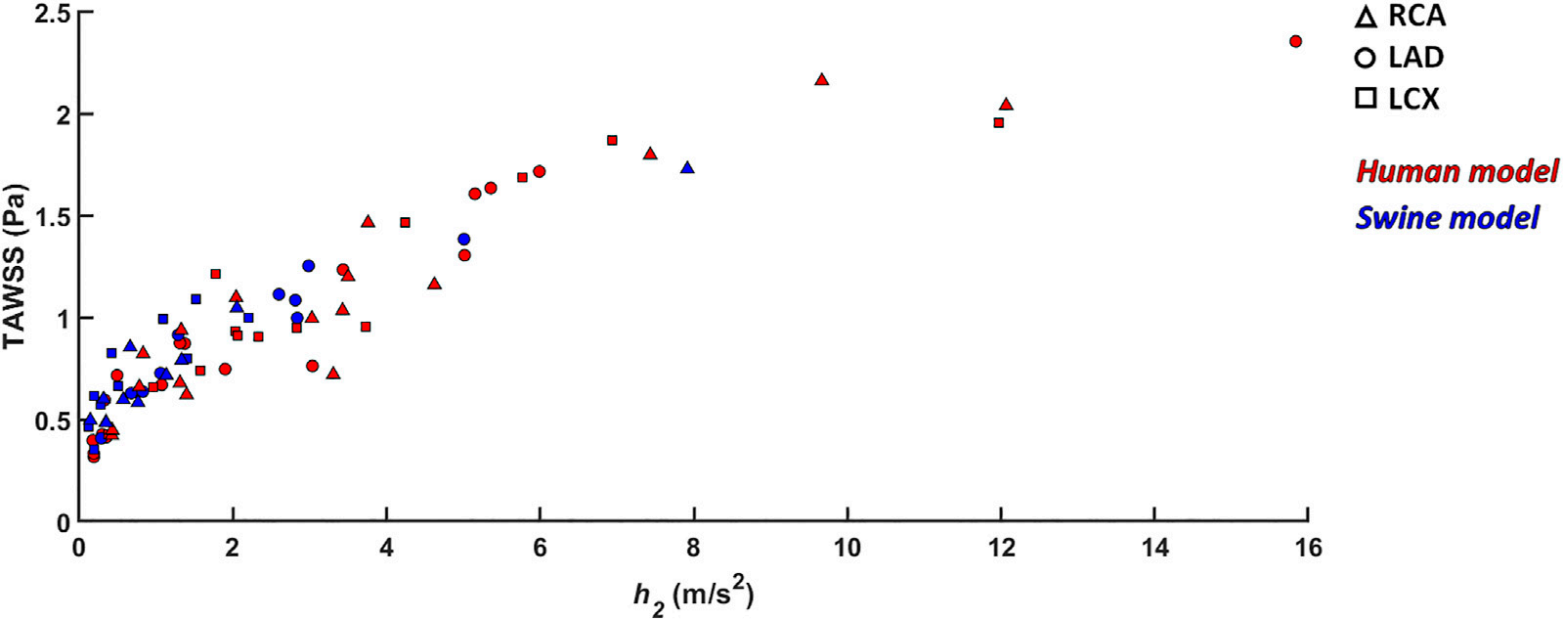
Scatter plots of helicity intensity *h*_*2*_ vs TAWSS. Red color indicates human models; blue color indicates swine models. Right (RCA), left anterior descending (LAD), and left circumflex (LCX) coronary artery geometries are distinguished by marker shape.

An overall inter-species equivalence emerged also in terms of intravascular hemodynamics, characterized by the presence of distinguishable counter-rotating helical flow patterns (Figures 5, 6). The evident similarity of helical flow features in human and swine coronary models finds confirmation in average helicity (*h*_*1*_), and in the balance between counter-rotating helical flow patterns (*h*_*3*_ and *h*_*4*_, respectively) in the three main coronary arteries (*Helicity* column in Figure 9). Significant inter-species differences emerged only for the helical flow intensity (*h*_*2*_), being higher in human RCAs (*p* < 0.05) and LCXs (*p* < 0.01) compared to the swine ones (*Helicity* column in Figure 9). However, despite the emerged significant difference in helical flow intensity, the relationship between the latter and WSS in coronary arteries, as previously reported for swine models (De Nisco et al., 2019), was also observed in human coronary arteries. Physiological high values of helical flow intensity keep TAWSS values within a physiological, atheroprotective range (Figure 10), and thereby prevent atherosclerotic plaque progression, which was also demonstrated by a direct association between plaque progression and helical flow intensity (De Nisco et al., 2020). Hence, the emerged inter-species difference in helical flow intensity does not cancel out but confirms previous findings on its physiological significance in swine coronary arteries (De Nisco et al., 2019, 2020), and remarks upon its possible use as surrogate marker of cardiovascular flow disturbances (Morbiducci et al., 2009, 2013; Gallo et al., 2012, 2015, 2018; Liu et al., 2015).

### Morphometry

The comparative analysis between human and swine coronary anatomical features confirmed the strong inter-species similarity. In addition to the already observed equivalence in vessel size and distribution (Weaver et al., 1986; Lelovas et al., 2014), human and swine coronary arteries are characterized by comparable values of mean curvature, mean torsion and tortuosity of the three main coronary arteries (*Morphometry* column in Figure 9). Interestingly, the emerged significant inter-species difference in coronary eccentricity ($\bar{\text{SI}}$), revealed that the luminal section of human coronary arteries is more elliptical than that of swine coronary arteries.

### Limitations

Several limitations could weaken the findings of this study. Computational hemodynamic modelling suffers from assumptions and uncertainties. Among them, the assumption of rigid vascular wall might affect WSS estimation. However, studies applying fluid-structure interaction approaches reported that WSS spatial distribution is preserved when using rigid walls (Torii et al., 2009; Malvè et al., 2012). Additionally, the cardiac-induced motion of coronary arteries was neglected. This idealization was based on previous evidences reporting the minor effect of myocardial motion on coronary flow and WSS distribution with respect to the blood pressure pulse (Zeng et al., 2003; Theodorakakos et al., 2008). Moreover, cardiac-induced motion could markedly affect instantaneous WSS distribution, but it has a minor effect on cycle-average WSS quantities as the ones considered in the present study (Torii et al., 2010). Finally, the limitations above affect swine as well as human populations. Hence, even not knowing whether their influence is species-independent, it might be negligible on the outcome of this study.

## Conclusion

Atherosclerosis is a multifactorial disease with hemodynamics as one of the main determinants of atherosclerotic plaque localization and progression. This study demonstrates that individual swine computational hemodynamic models of the three main coronary arteries are representative of the human hemodynamics in the same vessels. In detail, the study points out that swine and human coronary arteries present the same near-wall and intravascular hemodynamic features, as well as demonstrate anatomical similarities. These findings thus support the application of swine-specific computational models to investigate the hemodynamic-related risk of coronary atherosclerosis and have a high potential to translate directly into human coronary artery disease.

## Data Availability

The data analyzed in this study is subject to the following licenses/restrictions: the data can be shared upon request. Requests to access these datasets should be directed to j.wentzel@erasmusmc.nl.
